# Simplified speciation and atmospheric volatile organic compound emission rates from non‐aerosol personal care products

**DOI:** 10.1111/ina.12652

**Published:** 2020-02-26

**Authors:** Amber M. Yeoman, Marvin Shaw, Nicola Carslaw, Tim Murrells, Neil Passant, Alastair C. Lewis

**Affiliations:** ^1^ Wolfson Atmospheric Chemistry Laboratories University of York York UK; ^2^ National Centre for Atmospheric Science University of York York UK; ^3^ Department of Environment and Geography University of York York UK; ^4^ Ricardo Energy & Environment Gemini Building Harwell UK

**Keywords:** emission inventories, indoor air quality modeling, mass spectrometry, personal care products, siloxanes, VOCs

## Abstract

Volatile organic compounds (VOCs) emitted from personal care products (PCPs) can affect indoor air quality and outdoor air quality when ventilated. In this paper, we determine a set of simplified VOC species profiles and emission rates for a range of non‐aerosol PCPs. These have been constructed from individual vapor analysis from 36 products available in the UK, using equilibrium headspace analysis with selected‐ion flow‐tube mass spectrometry (SIFT‐MS). A simplified speciation profile is created based on the observations, comprising four alcohols, two cyclic volatile siloxanes, and monoterpenes (grouped as limonene). Estimates are made for individual unit‐of‐activity VOC emissions for dose‐usage of shampoos, shower gel, conditioner, liquid foundation, and moisturizer. We use these values as inputs to the INdoor air Detailed Chemical Model (INDCM) and compare results against real‐world case‐study experimental data. Activity‐based emissions are then scaled based on plausible usage patterns to estimate the potential scale of annual per‐person emissions for each product type (eg, 2 g limonene person^−1^ yr^−1^ from shower gels). Annual emissions from non‐aerosol PCPs for the UK are then calculated (decamethylcyclopentasiloxane 0.25 ktonne yr^−1^ and limonene 0.15 ktonne yr^−1^) and these compared with the UK National Atmospheric Emissions Inventory estimates for non‐aerosol cosmetics and toiletries.

**Practical Implications**

- Emissions of VOCs from the domestic sector, including personal care products, are highly uncertain, yet make up an increasing fraction of total VOC emissions in developed economies.
- The quantitative estimates of VOCs emitted from a range of personal care products provided here show that this information can constrain models of indoor air chemistry, particularly to make estimates of indoor concentrations of pollutants for which measurements are largely absent.
- Scaled estimated emissions provide a better guide to the contributions made by this source sector to personal emissions of VOCs than currently available and can be used to better understand personal exposure according to typical activities.

## 1 INTRODUCTION

Volatile organic compounds are a diverse class of air pollutants that, in high concentrations, can directly impact human health,^1^ and have widespread indirect effects through aiding the formation of secondary pollutants such as ozone and secondary organic aerosols (SOA). Both indoors and outdoors, VOCs are readily oxidized by O_3_ and radicals such as OH and can produce both tropospheric ozone and secondary organic aerosols when oxidized over several generations and in the presence of co‐pollutants such as NO_x_. The ability of SOA to scatter and absorb solar and terrestrial radiation, influence cloud formation, and participate in atmospheric chemical reactions means they play a significant role at scales beyond that of urban and regional air pollution.^2^ Additionally, as VOCs are a precursor to ozone and a sub‐component of PM_2.5_, they contribute to poor air quality and related health effects such as pulmonary inflammation and respiratory illness.^3^

From the 1970s onwards, global regulation and policy has focused primarily on reducing VOC emissions from sources such as the extraction and distribution of fossil fuels, combustion and leakage of fuels from road transport, natural gas networks, landfills, and coal‐fired power stations.^4^ Recently, as VOC emissions from fossil fuels and the transport sector have declined, the relative importance of other VOCs sources has increased.^5^ Historically, aims to regulate indoor VOCs tend to focus on building materials, and with particular attention toward compounds such as formaldehyde, benzene, and toluene. Less thought has been paid to the VOCs emitted from the use of PCPs (personal care products)^6^, ^7^, ^8^, ^9^, ^10^, ^11^, ^12^, ^13^, ^14^, ^15^ and HCPs (household cleaning products)^16^, ^17^, ^18^, ^19^, ^20^, ^21^, ^22^, ^23^ which, along with other domestic emissions of VOCs,^24^, ^25^, ^26^ are now known to be a substantial contributor to overall VOC emissions.^4^ Within this study, PCPs refer to cosmetic and hygiene products available to the public for personal use. PCPs are often split into two broad classifications for the purposes of VOC emissions reporting, described as non‐aerosol and aerosol, and it is non‐aerosol products that are reported here. The non‐aerosol class is potentially a smaller collective source of VOCs than aerosols, since the product matrix is often aqueous, whereas in the case of aerosol‐based PCPs, it is typically a hydrocarbon blend based around butane. Ethanol or oil‐based perfumes would be examples of PCPs based on hydrocarbons, although we do not test any of these in this study.

The mixture of VOCs emitted from sources such as gasoline evaporation is highly complex, but the detailed speciation of that source is reasonably constant and has been well‐characterized over time (see eg, Europe Environment Agency, emission inventory guidebook 2016^27^). Such mixtures are represented in some emissions inventories by an often complex speciation of VOCs, for example in the UK National Atmospheric Emissions Inventory.^28^ Air pollution models typically have a more simplified speciation, through combining (lumping) different VOCs into a smaller sub‐group of surrogate compounds, normally simple hydrocarbons, that are then explicitly treated subsequent oxidation mechanisms (see an overview of the topic in Carter, 2015^29^).

The situation is less well developed for consumer products, since each has a unique, generally proprietary, formulation and a substantial diversity in both speciation and emissions rates exists. To add to the complexity, many of the VOCs used in consumer products are high molecular weight and produce a range of multifunctional species when oxidized, some of which may be more harmful to health than the VOCs contained in the original product.^30^ For instance, the Master Chemical Mechanism, which is a near explicit mechanism developed to represent the degradation of VOCs in the atmosphere,^31^ needs 1244 reactions and 712 species to represent all of the reactions needed to go from limonene to the final oxidation products of water and carbon dioxide. This complexity means that representing their chemistry in models for indoor air chemistry is extremely challenging.

The ability to predict VOC emissions (both in terms of speciation and in absolute amounts) is needed however for management of indoor air quality, and to quantify the effects that domestic releases of VOCs have on outside air once ventilated. Nearly 90% of human exposure to VOCs is now believed to come from this kind of diffuse and largely unregulated set of sources that are within individual or household control, which includes consumer products,^7^ as well as other domestic sources such as glues, paints, sealants and other building products and materials. Other VOC sources in the home include natural gas leakage, pesticides, cooking, and combustion of wood, coal, and candles.^32^, ^33^

To understand our overall exposure to air pollution, it is vital to quantify the different sources of pollution both outdoors and indoors. In developed countries, we spend 80%‐90% of our time indoors and so our exposure to air pollutants, whether generated indoors or outdoors, will happen in the indoor environment. The use of PCPs is likely to represent a fraction of our overall exposure to pollution, but to date there has been little information available on how the use of an individual product could contribute to the emissions of VOCs, or the secondary products that can then be formed through subsequent chemical reactions. This knowledge requires detailed emissions measurements with sufficient speciation of the often complex formulations to understand the ongoing chemistry.

The estimation of VOC emissions rates from non‐aerosol PCPs is potentially a lengthy and time‐consuming process. Quantifying VOC content and emissions from PCPs using traditional methods such as headspace GC‐MS relies on the ability to predict the liquid‐gas partitioning of any given VOC, something that is virtually impossible to do given unknown formulations. Establishing whether an equilibrium has been reached between sample and the atmosphere above, it is difficult to achieve under realistic conditions with GC‐MS since the measurement frequency is rather slow, perhaps one measurement every 30 minutes. In a complex matrix where Henry's Law conditions likely do not apply, and where surface tension effects may be significant, a static headspace established over minutes to hours may not necessarily reflect VOC outgassing under more realistic non‐saturated dynamic conditions. The availability of fast responding on‐line mass spectrometry methods makes this a more tractable task in terms of tracking equilibration and VOC exchange, albeit with a penalty of less capability to speciate isomers and generally greater uncertainties in quantitative determinations. With on‐line methods such as proton‐transfer reaction mass spectrometry (PTR‐MS) and selected‐ion flow‐tube mass spectrometry (SIFT‐MS), the emission rate from a PCP sample can be tracked over minutes to hours using a dynamic flow of diluent gas over the sample and the temporal profile of concentrations then used to estimate the likely VOC emission rate and general VOC. The major advantage of using this method is that it has sufficient sensitivity for a direct analysis of a diluted dynamic headspace, avoiding the need for a pre‐concentration/thermal desorption step, and an equilibrium headspace concentration is typically determined in a few minutes. A limitation however of the method is that, like all online and direct inlet mass spectrometry methods, there is a more limited ability to differentiate between isobaric compounds, a notable issue if resolution between specific isomers (eg, monoterpenes or monoaromatics) is important. There are some advantages in terms of calibration using online MS, in that some reasonable first order estimate can be made of the concentrations of unknown VOCs in an unknown mixture, and without a primary standard available. But on‐line methods are inevitably less accurate than GC‐MS, if primary calibration mixtures for individual VOCs are available.

In this paper, the aim is to produce simplified emission profiles with a grouped speciation that are suitable for chemical models of indoor air and that can provide a guide to the scale of potential personal emissions of VOCs from this class of products. In turn, these values are then scaled upwards to place national emissions of VOCs from PCPs in context to other sources.

## 2 EXPERIMENTAL

### 2.1 Voice200 ultra SIFT‐MS

A Voice200 SIFT‐MS, by Syft Technologies, Christchurch, New Zealand, was used to identify and quantify VOCs emitted from a range of PCPs. The SIFT‐MS was operated with a flow tube temperature of 120°C, pressure of 460 mTorr, a voltage of 25 V, a sample flowrate of 25 sccm, and a Nitrogen (Research grade, BOC) carrier gas flow of 100 sccm which was maintained throughout the measurement period. The microwave ion source current was operated at 40 mW at 440 mTorr pressure.

**Figure 1:**
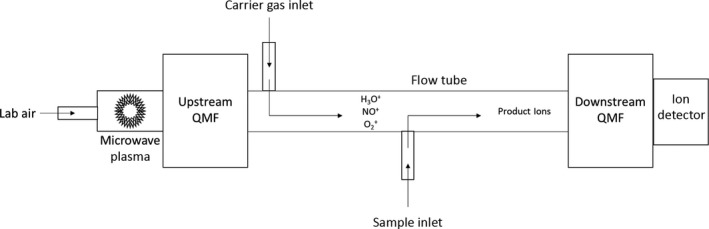
Schematic of the Selected‐Ion Flow‐Tube Mass Spectrometer instrument. QMF—quadrupole mass filter

A schematic outline of the Voice200 SIFT‐MS instrument is shown in Figure 1. The novel ion source region is where the reagent ions are generated in a microwave discharge, which acts on an air/water mix at a pressure of approximately 440 mTorr to generate the three reagent ions H_3_O^+^, NO^+^, and O_2_^+^. These ions are extracted into the upstream chamber maintained at a pressure of approx. 5 × 10^−4^ Torr. The reagent ions pass through an array of electrostatic lenses and the upstream quadrupole mass filter, and those not rejected by the mass filter are passed into the flow tube where they are carried along in a stream of nitrogen. The upstream quadrupole mass filter can rapidly (<1 ms) switch between the available reagent ions allowing a single measurement to use all available reagent ions essentially simultaneously.

### 2.2 VOC sampling

A total of 36 individual commercially available PCP samples were acquired from a UK supermarket. The objective was to sample a representative variety of products within each sub‐product class, covering a range of brands and formulations. The sample set used here comprised 7 shampoos, 9 shower gels, 12 moisturizers, 3 liquid foundations, and 5 conditioners. A small sub‐sample (500 mg) from each product was weighed and placed onto a small open vial, and spread to ensure a high surface area to depth ratio. The sub‐sample was then placed into a 10^‐ ^mL volume stainless steel gas‐tight sample vessel. The sample vessel comprised a stainless steel screw‐down lid and Viton O‐ring seal and two 1/16 in stainless steel Swagelok bulkhead connectors to provide an inlet and outlet for the diluent/sample gas. The stainless steel sample vessel containing the sample of PCP was thermostatted at 25°C for the first hour of the experiment (representative of ambient conditions) and 40°C for the second (to test whether VOCs could be completely driven off within a plausible user temperature envelope).

The samples were drawn into the SIFT‐MS at atmospheric pressure from the dynamic headspace of the stainless steel vessel at a flow rate of 25 mL min^−1^, with the inlet to the vessel connected to a VOC‐free supply of N_2_ gas. Before and after each PCP sample, an experimental nitrogen blank was carried out which was subsequently subtracted from each sample, although these VOC concentrations were typically very much smaller than the measured amounts, typically < 5%). For all the samples tested here, an equilibrium concentration of VOCs was established in the exiting gas, proportional to the amount of material under test and the VOC content. Over the temperatures and timescales of the testing, which are similar in nature to products in use, each sample acts as an approximately constant emission source of VOCs, and that emission rate is not appreciably changed through VOC depletion in the raw product. Over much longer timescales (hours to days) and/or higher test temperatures, then it is possible to drive off VOCs such that the emission rate declines until ultimately the VOCs are exhausted and emissions fall close to zero. For PCP use, we assume that VOC content in the mixture is not a limiting factor since both time and temperature fall within bounds of a few minutes and no more than 40°C. With that assumption, the amount of VOC released is then proportional to the amount of product used and the time that it is in use when VOC may evaporate. The assumptions we make here are tested against real‐world in‐use experiments.

Data on the VOC speciation and exact chemical makeup were acquired over a mass range of m/z 18‐400 using H_3_O^+^, NO^+^, and O_2_^+^ reagent ions separately. The suite of selected masses was measured with a dwell time of 0.1 seconds per m/z which resulted in a total measurement cycle of 38.3 seconds. Data acquisition lasted for 120 minutes per sample which provided ~60 mass spectra for each reagent ion for sample averaging. Data acquisition and processing was carried out using the instrument Labsyft software.

### 2.3 Data analysis

Measured product ions were normalized (for both blank and samples) by dividing the identified product ion intensities by the sum of their reagent and their respective water cluster ion intensities. These were H_3_O^+^: (m/z 19), H_3_O˙ H_2_O^+^ (m/z 37), H_3_O˙ H_2_O^+2^ (m/z 55), and H_3_O˙ H_2_O^+3^ (m/z 73), NO^+^: (m/z 30), and NO˙H_2_O^+^ (m/z 48), O_2_^+^: (m/z 32), and O_2_˙ H_2_O^+^ (m/z 50). To simplify the data analysis, only the most intense 30 ion signals from each reagent ion reaction were then selected for further data processing. It should be appreciated that the most highly emitted compounds on a mass basis may not hold the most significance in terms of their relative health implications and reactivity; however, for the purpose of reporting a simplified speciation profile for personal exposure the data has been selected in this way. It is worth noting that for the purposes of reporting emissions of VOCs under transboundary treaties, a mass‐based metric is still used, rather than on VOC reactivity or downstream impact.

To allow for a simple visualization of the key VOC emissions from all PCP samples, tile plots were constructed. These give an overview of the most abundant product ions found in each sample with product ion intensity, displayed as the color scale. Some known fragmentation and product ions of monoterpenes have been removed to simplify data visualization, leaving m/z 137 and 151 to represent the H_3_O^+^ product ions, m/z 136 and 154 for NO^+^, and 93 for O_2_^+^. All monoterpenes considered are represented by at least one of these ions. There is confidence that none of the removed ions represent parent compounds other than monoterpenes. All product and fragment ions were identified using the Labsyft software. Further details of the methodology for monoterpene fragmentation and product ion identification are given in Table S1. On a small number of occasions where samples contained major VOC ions in the SIFT‐MS that could not be directly identified or attributed to a given VOC class, like monoterpenes, we used a confirmatory GC‐MS (Agilent 6890‐5973) analysis to provide us with further information in toward an identification.

### 2.4 Atmospheric model

The model used in this paper is the INdoor air Detailed Chemical Model (INDCM) described in detail by Carslaw^34^ and Carslaw et al.^35^ Briefly, the model uses the Master Chemical Mechanism (MCM), v3.2.31,^36^, ^37^, ^38^ which treats the degradation of VOC near‐explicitly from the initial oxidation step by the hydroxyl radical, the nitrate radical, ozone, or photolysis as relevant and then follows the products of these reactions until carbon dioxide and water are formed as the final oxidation products. The chemical mechanism is then coupled with terms that deal with exchange of pollutants between indoors and outdoors, deposition to internal surfaces, internal emissions, and photolysis (both from attenuated outdoor light and from artificial lighting indoors). The model can be parameterized to be any indoor space (eg, office, bathroom, classroom) and in any geographical location. External pollutant concentrations can then be set as appropriate. The model assumes the internal environment is well‐mixed.

For the purposes of this study, the model was set to simulate an en suite bathroom in order to simulate the use of PCPs during a shower. The bathroom was assumed to have dimensions of 1.55 × 1.8 × 2.1 m giving a volume of 5.5 m^3^. In order to calculate the overall area to volume ratio for the bathroom, we calculated the area of the floor (2.55 m^2^) and the walls (13.65 m^2^) and then weighted each according to their typical area/volume ratios as defined in Kruza et al.^39^ This gave an overall area to volume ratio of 0.01 cm^2^/cm^3^. We used a ventilation rate for bathrooms of 50 m^3^ h^−1^ based on a range of values in European residences (Dimitroulopoulou^40^), which provides 9 air changes per hour (ACH). We have also assumed a relative humidity of 70% (based on Laverge et al^41^) and temperature of 293 K.

External pollutant concentrations were typical for a polluted European city such that outdoor ozone, nitric oxide, and nitrogen dioxide mixing ratios were ~24, 20, and 23 ppb, respectively, during the period we show in the Results section (07:00‐08:30 h). These external concentrations produced internal mixing ratios for these three pollutants of ~11, 8, and 31 ppb, respectively, for the same period in the absence of any showering activities. External VOCs were as described by Kruza et al^39^ and were used to drive the indoor chemistry in the absence of indoor activities.

Out of the seven common VOCs identified in the samples in the previous section, methanol, ethanol, 2‐propanol, benzyl alcohol, and limonene are already represented in the chemical mechanism (the MCM) within the INDCM. The D4 and D5 siloxanes are not included. Based on the literature, the fate of the siloxanes outdoors is to react with the OH radical and deposition is relatively unimportant.^42^ Indoors, there is a relatively large surface area available and lower OH concentrations, so the relative importance is likely to be different. Therefore, reactions of D4 and D5 with OH were added to the model mechanism, with rate coefficients of 1.01 × 10^−12^ and 1.55 × 10^−12^ cm^3^ molecule s^−1^, respectively.^43^ These reactions were assumed to form silanols.^42^, ^44^ Whelan et al^45^ suggested that the cVMS (cyclic Volatile Methyl Siloxane) species had a dry deposition velocity of 0.3 cm s^−1^ outdoors and also that the silanols were more likely to undergo deposition than the parent siloxanes. Based on the method described by Carslaw et al,^35^ the outdoor deposition velocity was divided by 20 to provide an indoor equivalent of 0.015 cm s^−1^ for the cVMS species. We then doubled this value (0.03 cm s^−1^) to estimate a deposition velocity for the thiols.

The limonene measured in this study represents the sum of all monoterpenes. For the purposes of modeling, we treat this mechanistically as limonene, but denote it our results as limonene* in recognition that our model is not predicting for limonene exclusively. Although there are differences in chemistry between different monoterpenes in terms of rate coefficients for reaction with OH, O_3_ and NO_3_ and also yields of radical production, it is the most ubiquitous and abundant monoterpene measured indoors^46^ and so this simplification seems reasonable for the purpose of this study.

## 3 RESULTS AND DISCUSSION

### 3.1 Estimation of emission rates

The SIFT‐MS is used to measure the time‐dependant concentrations of VOCs in the dynamically flowing headspace passing over the sample. By following this concentration over a period of two hours to a continuous equilibrium value, it is implied in all cases that there is no limitation on available VOC for evaporation for the duration of the test (and at test temperature). By knowing the sample flow rate (typically ~10 mL min^−1^), an estimate was then made for individual VOC emission rates from each PCP. Since no information exists a priori for the speciation of the VOCs in each sample, the calibration and quantification of each VOC relies on the internal instrument/software estimation of concentration made via H_3_O^+^, NO^+^, and O_2_^+^ reaction kinetic parameters in the MS ionization source. Where it has not been possible to directly calibrate individually for specific compounds, we assume an absolute uncertainty of 20%‐25%, based on our own laboratory measurements and as reported in other publications using this instrument.^47^, ^48^ For some species, we are able to directly calibrate the SIFT in the laboratory based on gas standards and so have a good understanding of instrument response factors, for example for ethanol, aromatic hydrocarbons, and limonene. For other VOCs, we do not have a primary gravimetric standard but can estimate factors such as relative transmission efficiency and fragmentation patterns, for example for siloxanes, based on stable working mixtures that can be blended over different concentrations ranges and instrument operating conditions. Concentration data for each product is available in Table S2. Experiences of using first‐principles calibration with other types of PTR‐MS instruments suggest the uncertainty could be larger than this, although set in context with the potential uncertainties in the usage scenarios, if a wider uncertainty calibration range is used it does not materially change any conclusions reported here.

**Figure 2:**
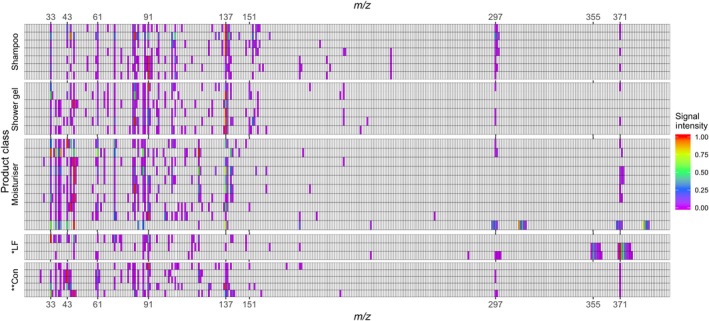
Visualization of VOC emissions from 36 different PCPs based on H_3_O^+^ ionization. Data from each PCP sample is normalized to the maximum product ion intensity in that sample. Fragment ions are removed. *LF—liquid foundation, **Con—conditioner

Figure 2 shows the relative VOC emissions rates by product ion for the H_3_O^+^ reagent for each of the different PCPs. The product ion intensities for each PCP dataset are normalized to the highest product ion. While it is clear that each sample is unique in terms of its VOC speciation, common “bands” of species do emerge across the sample types. The tile plots generated from the NO^+^ and O_2_^+^ ions are shown in the Figures S1 and S2), but they indicate a similar speciation to that from H_3_O^+^, albeit with different individual ion intensities reflecting differing ion chemistries.

From this analysis, a simplified emission profile based on seven most common and abundant individual VOCs is proposed, lumping in cases where isobaric overlaps exist, and/or where the data does not allow for speciation, for example among different monoterpenes. The simplified PCP VOC speciation comprises methanol, ethanol, 2‐propanol, benzyl alcohol, limonene (representing the sum of monoterpenes), D4 (Octamethylcyclotetrasiloxane), and D5 (Decamethylcyclopentasiloxane).

**Figure 3:**
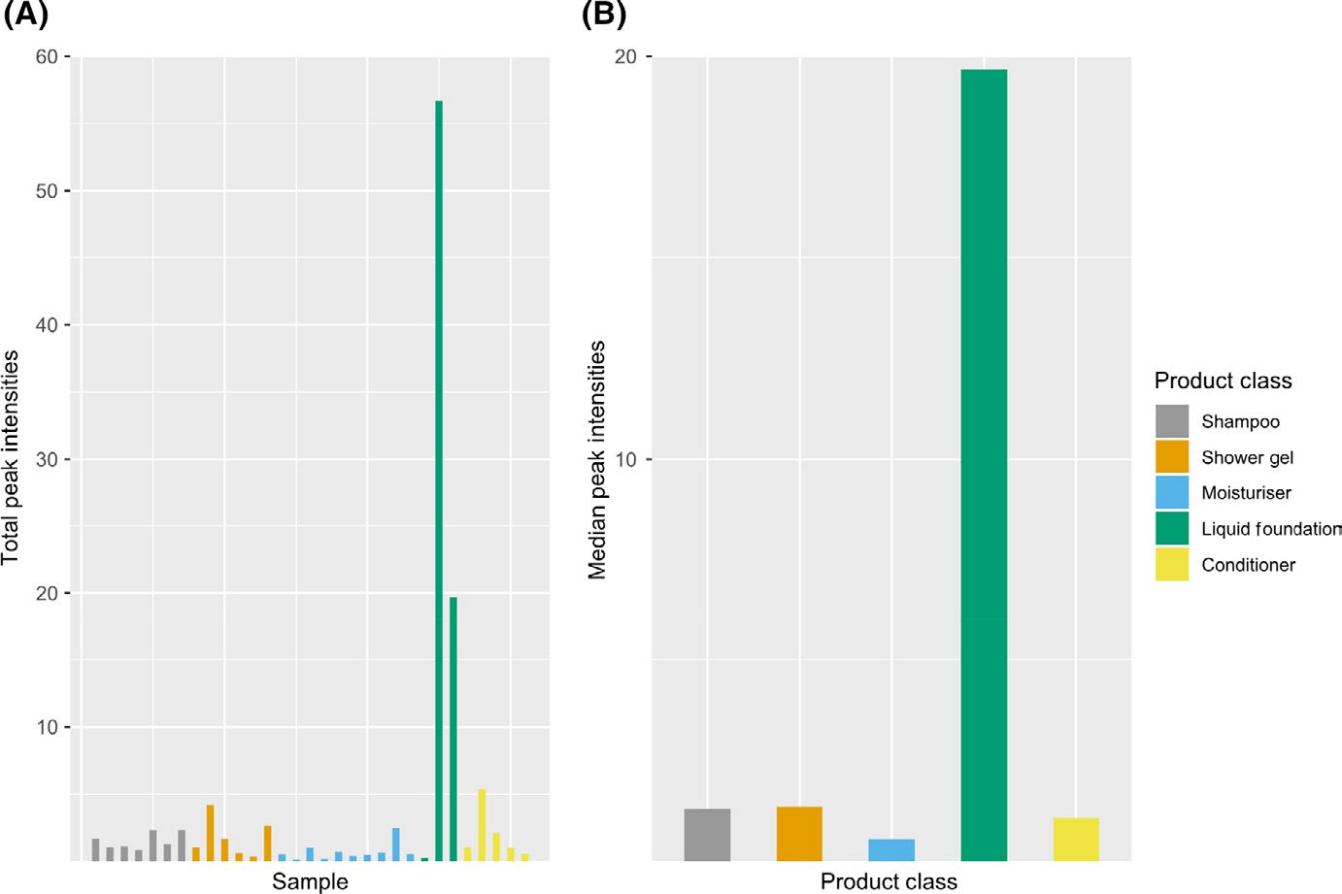
A, Summation of total VOC product ion peak intensities for each PCP tested and B, median emission intensity for each product class

A simplified overview of total VOC emission from each product can be gained from examining the relative differences in total ion count (when normalized for reagent ion amounts) for each sample. This provides a basic indication of how variable VOC emissions rates are both between and within PCP product classes. The 30 largest product ion signals from each of the three different reagent ions, including all fragmentation and product ions, were summed (eg, giving 90 ions in total), to provide a total VOC product ion count, taken as a proxy for overall VOC emission rate by mass. It should be stressed that this is essentially an arbitrary unit and cannot be directly transferred as an absolute mass of carbon emissions, but it is helpful in understanding how variable emissions rates are between products. This is shown for the various sample classes in Figure 3 as total peak intensity for each PCP under test along with the median value for each class of PCP.

Highest total VOC emissions were associated with liquid foundation, predominantly due to very high emission rates of cVMS D4 and D5. Within the individual product classes, total VOC emission rates varied considerably, often by more than an order of magnitude, suggesting that a very specific level of VOC content is not a fundamental pre‐requisite in the formulation of these products.

The emission of VOCs from non‐aerosol PCPs is potentially complex, since it is likely that only a fraction of the overall VOC content in each sample is released in the room where the product is used. Our approach is to estimate the emission rate as a function of amount of product and time in use. For wash‐off products, some fraction of the VOC content of the product is not released, but instead remains in the aqueous phase in dilute amounts, washed away. The fate of this fraction of the VOC is essentially unknown. Within our calculations, we assume the only VOC emissions are those which occur during the direct product use in‐room. It is possible that VOCs also escape to the air at some later stage, for example from waste‐water, but we do not attempt to account for this in the scale‐up calculations. For leave‐on products such as moisturizers and liquid foundation (which remain on the skin, not washed off) more time is potentially available for VOCs to evaporate to air compared to wash‐off products. Here, longer “in‐use” scenarios are probably appropriate, but these must have some upper bound since the amount of VOC in the product is finite. We chose to express the individual VOC emissions as a mass released per unit time per gram of product and then, in a later section, apply an in‐use period to each product. For example, one scenario is that a shower gel unit of activity may comprise a 4 g PCP sample in use for 30 seconds. Such an approach has to assume that as for the laboratory equilibrium determinations, over the actual periods of PCP activity/usage, the VOC liquid phase concentrations are not a limiting factor for VOC transfer to the gas phase, but rather the limitation is the mass transfer of VOC out of the product as a vapor. Table 1 shows the calculated emission factors from the simplified emission profiles as a function of time and mass of product at 25 ℃.

**Table 1:** Estimated product emission factors at 25°C for each non‐aerosol PCP type using a simplified VOC emission profile

	PCP in‐use Emission Factors (μg s^−1^ g_[product]_^−1^)						
	2‐Propanol	Benzyl Alcohol	D4	D5	Ethanol	Limonene	Methanol
Shampoo							
1	1.1	2.1 × 10^−1^	2.6	2.6 × 10^−1^	7.6 × 10^−2^	5.9 × 10^1^	9.6 × 10^−2^
2	3.7 × 10^−1^	1.2	4.0	4.2 × 10^−1^	3.5 × 10^−1^	9.0	4.3 × 10^−1^
3	1.4 × 10^−1^	2.8 × 10^−1^	1.9	2.7 × 10^−1^	1.3 × 10^−1^	2.6 × 10^1^	2.2 × 10^−1^
4	7.3 × 10^−2^	8.7 × 10^−2^	2.2	2.8 × 10^−1^	2.6 × 10^−1^	2.5 × 10^1^	6.4 × 10^−1^
5	1.2	5.8 × 10^−1^	2.1	4.6 × 10^−1^	1.8 × 10^−1^	1.6	1.3
6	6.0 × 10^−2^	4.1 × 10^1^	1.6	3.5 × 10^−1^	7.5 × 10^−2^	7.1	1.8
7	1.4 × 10^−1^	9.5 × 10^−1^	2.0	4.4 × 10^−1^	4.7 × 10^−1^	7.0 × 10^1^	1.8 × 10^−1^
Median	1.4 × 10^−1^	9.5 × 10^−1^	2.1	3.5 × 10^−1^	1.8 × 10^−1^	2.5 × 10^1^	4.3 × 10^−1^
Shower Gel							
1	1.2 × 10^−1^	1.8 × 10^1^	—	—	2.5 × 10^−1^	4.4	1.7
2	3.3	6.9 × 10^−1^	—	—	6.0 × 10^−1^	1.2 × 10^2^	1.0 × 10^1^
3	7.2 × 10^−2^	6.9 × 10^−2^	—	—	1.0 × 10^1^	1.5 × 10^1^	7.9 × 10^−2^
4	4.7 × 10^−2^	6.5 × 10^−1^	—	—	1.6 × 10^−1^	6.2	2.6 × 10^−1^
5	8.0 × 10^−2^	2.5 × 10^−1^	—	—	7.4 × 10^−2^	1.4	8.3 × 10^−2^
6	5.9 × 10^−2^	3.9 × 10^−2^	—	—	9.4 × 10^−2^	4.2 × 10^1^	1.9 × 10^−1^
Median	7.6 × 10^−2^	4.5 × 10^−1^	—	—	2.4 × 10^−1^	1.1 × 10^1^	2.3 × 10^−1^
Moisturizer							
1	3.2	5.2 × 10^−2^	—	3.1 × 10^−1^	6.8 × 10^−1^	1.4	4.2 × 10^−1^
2	2.5 × 10^−2^	2.2 × 10^−2^	—	2.1 × 10^−1^	1.0 × 10^−1^	5.2 × 10^−2^	1.3 × 10^−1^
3	2.9 × 10^−1^	2.5 × 10^−2^	—	2.2 × 10^−1^	8.5	2.8	2.5 × 10^−1^
4	2.1 × 10^−2^	2.7 × 10^−2^	—	3.0 × 10^−1^	3.0 × 10^−1^	3.1 × 10^−1^	4.8 × 10^−2^
5	8.7 × 10^−2^	1.1	—	2.6	3.2	2.4	2.2 × 10^−1^
6	4.0 × 10^−2^	1.2 × 10^−1^	—	2.1 × 10^−1^	6.0 × 10^−2^	7.6 × 10^−1^	8.7 × 10^−2^
7	7.2 × 10^−2^	1.0 × 10^−1^	—	7.1 × 10^−1^	3.2	2.9 × 10^−2^	1.2
8	4.4 × 10^−1^	1.3	—	3.5 × 10^−1^	1.1 × 10^−1^	5.8	8.8 × 10^−2^
9	1.0	1.9 × 10^1^	—	4.6 × 10^−1^	1.1 × 10^−1^	1.1 × 10^−1^	2.3 × 10^−1^
10	6.6 × 10^−1^	6.7	—	5.7 × 10^−1^	3.5 × 10^−1^	1.6 × 10^−1^	1.8 × 10^−1^
Median	2.0 × 10^−1^	1.1 × 10^−1^	—	3.3 × 10^−1^	3.3 × 10^−1^	5.3 × 10^−1^	2.0 × 10^−1^
Liquid Foundation							
1	—	—	—	2.7 × 10^−1^	6.4 × 10^−1^	1.6 × 10^−2^	—
2	—	—	—	6.8 × 10^2^	7.8 × 10^−1^	3.0 × 10^−1^	—
3	—	—	—	7.0 × 10^2^	4.3 × 10^−2^	1.9 × 10^−2^	—
Median	—	—	—	6.8 × 10^2^	6.4 × 10^−1^	1.9 × 10^−2^	—
Conditioner							
1	6.6 × 10^−1^	1.5 × 10^1^	5.7 × 10^−1^	1.8 × 10^1^	5.1 × 10^−2^	5.2 × 10^−1^	2.1 × 10^−2^
2	7.9 × 10^1^	3.7	1.5	5.9 × 10^−1^	5.4 × 10^−2^	3.3	2.1
3	3.7 × 10^1^	1.0 × 10^−1^	1.4	5.8 × 10^−1^	4.4 × 10^−2^	1.6	9.1 × 10^−1^
4	2.8	6.3 × 10^−1^	7.4 × 10^−1^	4.5 × 10^−1^	7.2 × 10^−2^	7.6	6.3 × 10^−1^
5	5.9 × 10^−2^	1.1 × 10^−1^	1.1	3.6 × 10^−1^	2.6	1.5	7.2 × 10^−1^
Median	2.8	6.3 × 10^−1^	1.1	5.8 × 10^−1^	5.4 × 10^−2^	1.6	7.2 × 10^−1^

Since the range of total VOC emissions found in each product class is highly variable, for the subsequent calculations we report the median emissions of each VOC within each of the PCP classes.

The values in Table 1 provide a starting point for possible explicit modeling of the effects of PCP VOC emissions, although further parameters require defining if an overall mass of emission of any given VOC is to be estimated. Using emissions factors on a per unit time and mass of product basis assumes that VOC emissions will scale linearly with additional time that they are in use (exposed to air) and additional mass of product used, up to some total maximum emissions limited by the VOC amount in the PCP dose. We develop here a range of scenarios for each PCP when in use. These various in‐use scenarios are then used to scale‐up the activity data to a per‐person annual estimate of emissions for each product and then scaled further to give an indication of the potential scale of contribution of this source type at a national scale, using the United Kingdom as an example.

There is limited literature guidance on typical in‐use scenarios, so we must use our own best‐estimates of a plausible range. The range of these scenarios (meaning amount of product used and time‐scale for use) is such that this in turn creates a wide range of potential VOC emissions, something that could only be narrowed if more precise information on PCP in‐use activity was available to us. For our estimates, shampoo usage is assumed to be proportional to that of conditioner. Moisturizer is the most difficult product class to estimate, as many products fall into this category and are used in a variety of ways, both in terms of amount and frequency (eg, a small amount of eye cream is used daily compared to multiple hand cream applications), and it therefore has the largest estimated range of in‐use emissions.

### 3.2 Annual estimates of emissions of VOC from non‐aerosol PCPs

**Figure 4:**
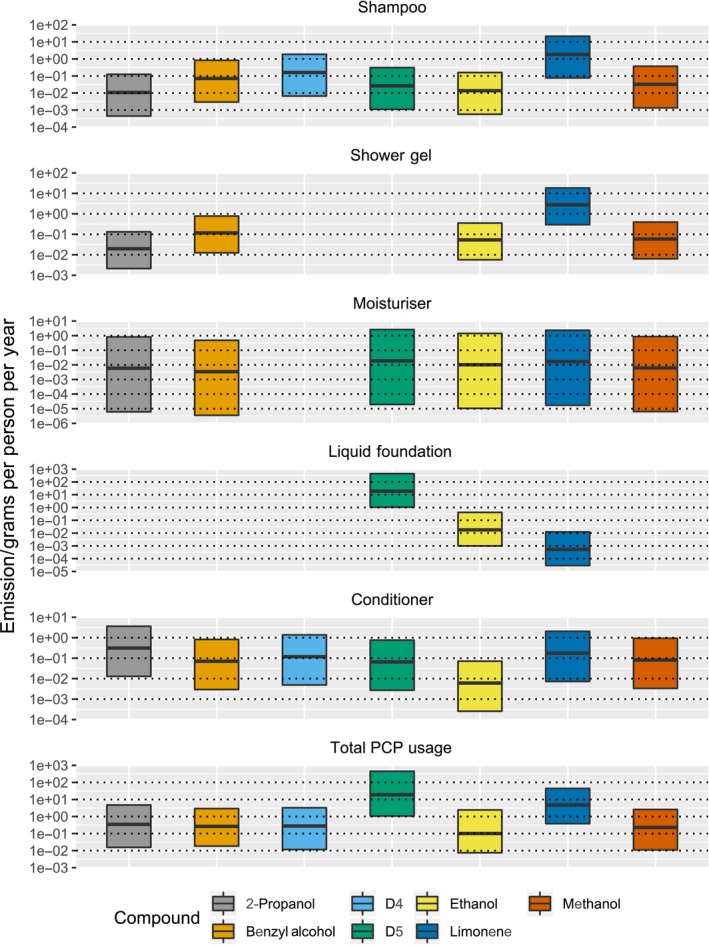
Range of potential VOC emissions from various non‐aerosol personal care products on an annualized basis covering three activity and frequency scenarios outlined in Table 2

The laboratory measured emissions factors are combined with the range of activity scenarios in Table 2 to produce a simplified set of potential annual emissions statistics of VOCs from PCPs on a per‐person basis. For each PCP class, we have taken the median VOC emissions from the group of products tested. This median emission is then scaled by the three usage scenarios, to give a lower and upper bound and central estimate value for annualized per‐person emissions as in Table 2. Table S3 provides the summary of emissions for each product type and for the seven VOCs in the simplified VOC profile. We show this data in graphical format in Figure 4 for each of the products and for each of the seven VOCs within the simplified profile.

**Table 2:** PCP in‐use consumption scenarios/activity levels later applied to individual emission factors for each product (L—low, M—medium and H—high.)

Product Class	PCP Used in each Unit of Activity (g)	Period of Use (s)	Unit Activity (s g)	Annual Frequency of Activity (yr^−1^)
Shampoo				
L	2	30	60	52
M	4	120	480	156
H^a^	8	300	2400	364
Shower Gel				
L	3	60	180	156
M	4	180	720	364
H^a^	8	300	2400	728
Moisturizer				
L	0.5	5	2.5	13
M	5	120	600	52
H^a^	10	600	6000	728
Liquid Foundation				
L	2	60	120	13
M	3	180	540	52
H^a^	6	300	1800	364
Conditioner				
L	3	30	90	52
M	6	120	720	156
H^a^	12	300	3600	364

a High scenario taken as the complete release of all VOCs contained in each product based on experimental estimates of emissions.

The seven species are selected to represent a simplified speciation based on data from Figure 2.

### 3.3 Comparisons against emission inventory estimates

The reporting of VOC emissions forms part of obligations for signatories to the UN‐ECE Convention on Long‐Range Transport of Air Pollution (CLRTAP, see: http://www.unece.org/env/lrtap/welcome.html.html), where a country is required to provide estimates of annual emissions for the purposes of demonstrating compliance with ceiling targets. Similar obligations exist in the EU under the National Emissions Ceiling Directive (NECD). Reporting of emissions is however generally expressed as a single national total tonnage, and the degree to which speciation of VOCs is available (by compound and emitting sector) in different countries is highly variable. The NECD and CLRTAP defines those VOC sources to be included and excluded from the national inventory, notably VOCs from biogenic sources are excluded, and provides the technical definition of “what is a VOC.” They also define how emissions from different sources are categorized between emitting sectors such as energy, transport, industrial and so on.

The EEA/EMEP Guidebook provides estimation methodologies and default emission factors for each source category. Country‐specific emission factors can be used where deemed relevant, which may be the case for industrial process emissions, but less so for common sources such as road transport. However, although the reporting of VOCs appears very detailed, the methodologies used are heavily skewed to the dominant sources of VOC emissions as found in the late 1980s and 1990s, the time these treaties and methodologies were being developed. At that point, the overwhelming source of VOCs to air was from fuels and transportation and it is understandable that relatively little emphasis was placed at that time on reporting in detail VOCs from consumer products.

Few countries provide estimates of VOCs emission at a level of speciation, activity, and source sector granularity that would allow for comparison against data of the kind provided in this study. The United Kingdom National Atmospheric Emissions Inventory (NAEI) is possibly the most detailed national emission reporting system of VOC found globally and attempts to make some estimate of emissions of VOCs from sources such as personal care products (NAEI, 2019^49^). The NAEI includes more than 2000 different sources of VOCs and in excess of 600 different VOCs are included. The methodology is described in Passant NR 2002.^28^

Using a mid‐year 2017 estimate of the UK population of 66 million people^50^ some extrapolation of potential national annual emissions of VOCs from non‐aerosol PCPs can be made. Of course, to scale further from our per‐person estimates carries with it the wide range of scenarios in Table S3 providing ultimately a very broad range of potential emissions. Nonetheless, it is potentially useful to place those bottom‐up estimates of emissions against the emissions currently included for this source class within the UK NAEI. Table 3 shows the activity and frequency scenarios then scaled for the UK as a whole, but with the application of some de‐ratings to reflect that not all of the population will be users of each of those product types. We apply a reduction factor of 0.8 to shampoo and shower gel, 0.4 to conditioner, 0.25 to moisturizer, and 0.2 to foundation. For comparison, we then extract from the 2017 UK NAEI the VOC emissions estimated under the EEA/EMEP Guidebook categorization of “Solvent Use,” sub‐class “Non‐aerosol Products – Cosmetics and Toiletries,” NFRCode: 2D3a and Source Code: 256.

**Table 3:** Estimated annual UK VOC emissions from non‐aerosol PCP use and comparison with 2017 UK NAEI estimates for the “Non‐aerosol Products – Cosmetics and Toiletries” class of emissions. Calculations based on all UK users (following a de‐rating to account for non‐users) being either high, medium, or low emissions as set out in product‐use scenarios in Table 2)

Compound	Low (kg yr^−1^)	Medium (kg yr^−1^)	High (kg yr^−1^)	UK annual emissions NAEI (kg yr^−1^)
2‐Propanol	4.6 × 10^2^	9.4 × 10^3^	1.2 × 10^5^	3.1 × 10^4^
Benzyl Alcohol	7.4 × 10^2^	8.1 × 10^3^	7.1 × 10^4^	5.2 × 10^1^
D4	1.3 × 10^2^	3.1 × 10^3^	3.6 × 10^4^	0
D5	1.4 × 10^4^	2.5 × 10^5^	5.9 × 10^6^	0
Ethanol	3.2 × 10^2^	3.4 × 10^3^	5.0 × 10^4^	2.1 × 10^7^ a
Limonene	1.6 × 10^4^	1.5 × 10^5^	1.1 × 10^6^	0
Methanol	4.2 × 10^2^	6.2 × 10^3^	6.0 × 10^4^	0

a The national estimate for ethanol within the cosmetics and toiletries category includes perfume which represents the bulk of estimated ethanol emissions in this class.

The most immediate observation to be drawn from Table 3 is that of the seven major VOCs found in PCPs, four of these do not currently have any emissions included in the NAEI for this source classification (although all are included in the NAEI and emitted from other sources). Bottom‐up extrapolation would suggest that for the central estimate of activity and usage, D5 cVMS (0.25 ktonne yr^−1^) and limonene (0.15 ktonne yr^−1^) are the most significant national VOCs by mass of emissions arising from non‐aerosol PCP use. For perspective, however, the overall UK emission total for VOCs was estimated at 807 ktonne for 2017 (of which 579 ktonne were solvents), so this VOC contribution from non‐aerosol PCPs to overall national emissions is very modest. The significance as a perturbation to indoor air quality where the concentrations would be maximized is explored further in the next section.

### 3.4 Model simulations

**Figure 5:**
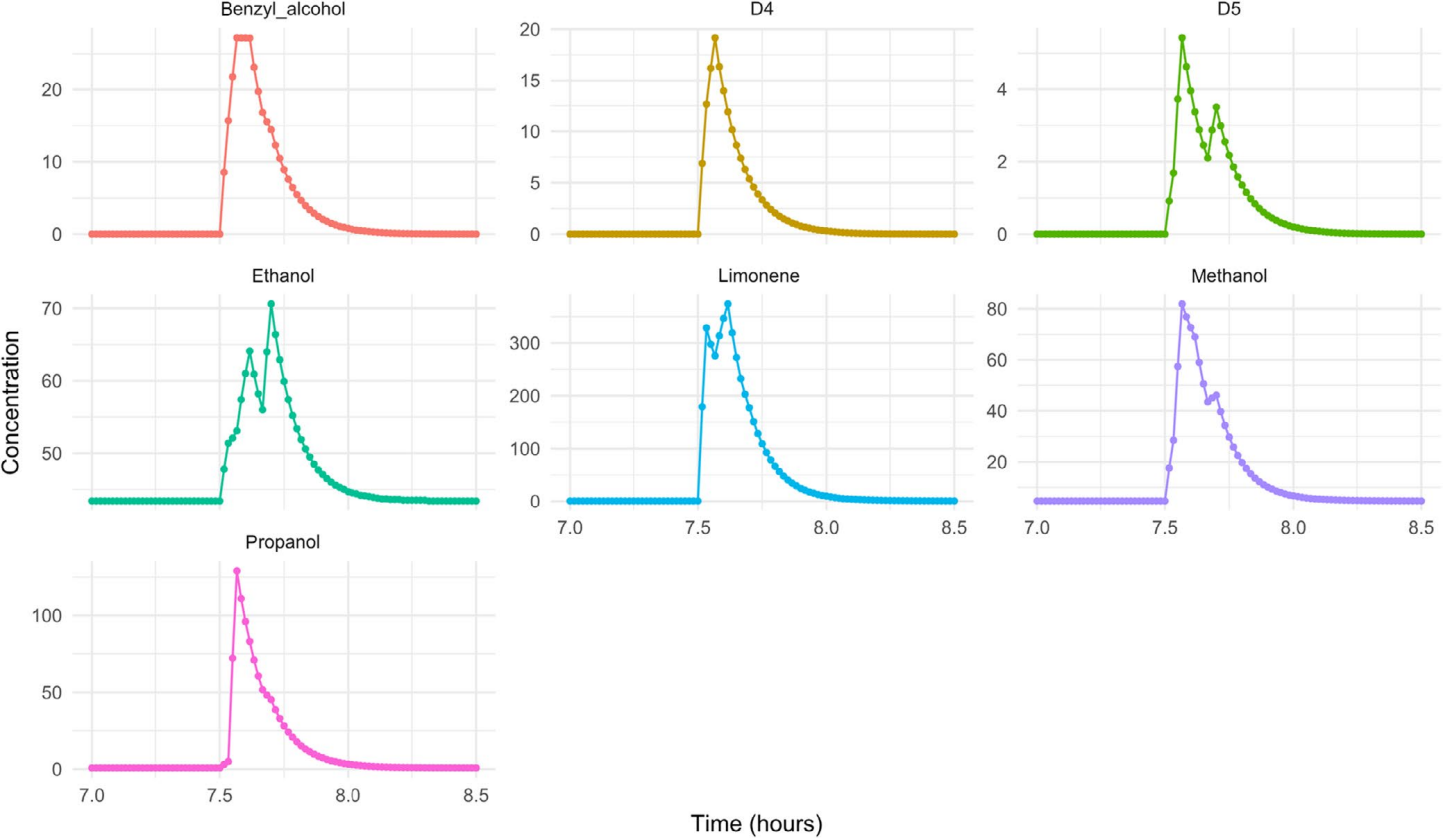
Mixing ratios of the seven components of the PCPs investigated following a shower using shampoo, conditioner, shower gel, and moisturizer afterward (units ppb)

The emission rates from Table 1 were used to explore ambient concentrations that could arise following a representative use of PCPs within a shower. The median values were used for each of the seven VOCs/VOC classes, and the activity was assumed to be as follows. The shower commenced at 07:30 h, with the first 2 minutes spent using shampoo, followed by 2 minutes using conditioner and a further 3 minutes using shower gel. It was then assumed that there was a 3‐minute pause to dry off, followed by 2 minutes spent applying moisturizer. The model was then used to explore the mixing ratios that could arise following the shower. Figure 5 shows the concentrations of the primary emissions based on Table 1 and focusing on the period from 07:00 to 08:30 hours.

Figure 5 shows that the mixing ratios of the primary emitted species increase as the shower begins and attain high concentrations, even at the relatively high ventilation rate of 9 h^−1^. The profiles for the different species show differences according to their emission rates from the different processes, for instance, there is no D4 or D5 in the shower gel, whereas limonene* is present in all the PCPs used. Even though PCP use is only from 7:30‐7:39, elevated concentrations are sustained beyond this period. Note that under these conditions, limonene* mixing ratios peak at ~375 ppb.

**Figure 6:**
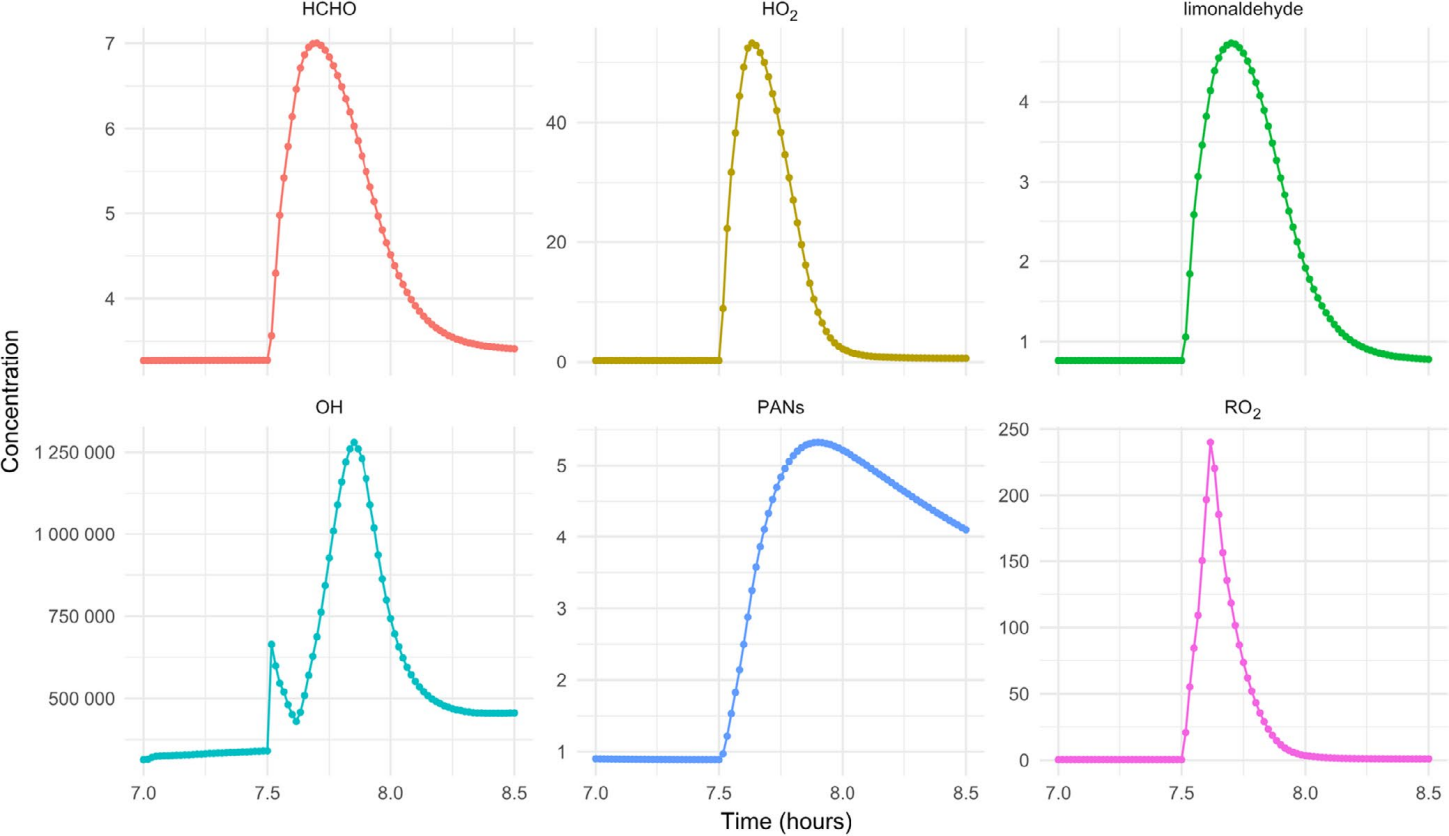
Concentration of OH (units molecule cm^−3^) and mixing ratios of HCHO, limonaldehyde and PANs (sum of all the PAN‐type species in the model) in ppb and HO_2_ and RO_2_ in ppt

Figure 6 shows some of the species formed through the chemistry. Despite the high concentrations of limonene*, the high ventilation rate limits the potential for secondary chemistry, and formaldehyde and limonaldehyde (oxidation products of limonene) concentrations are only enhanced by about 4 ppb during showering, though they are still slightly elevated an hour or so afterward. Concentrations of PAN‐type species in the model are elevated by ~4 ppb during the shower, but higher mixing ratios are sustained for longer than the other secondary species, owing to their much longer lifetimes under these conditions. Fine particle concentrations (not shown) were enhanced by around 1 µg/m^3^ as a result of the PCP use, owing to the propensity of limonene oxidation products to form particulate matter.^51^ Figure 6 also shows the impact of showering on the temporal evolution of the radical concentration. The OH concentration is enhanced as ozonolysis of limonene produces OH radicals, causing the concentration to peak at about 1.3 × 10^6^ molecule cm^−3^, with HO_2_ and RO_2_ mixing ratios peaking at 50 and 240 ppt. The OH concentrations are typical for those you might expect outside and show that conditions indoors can lead to significant quantities of radicals, even in the absence of sunlight. The peroxy radical concentrations are enhanced relative to those typically observed outside.^52^ Note that some of these species are water‐soluble gases^53^ and may dissolve in water during showering. These processes are not currently included in the INDCM, so the values we present are likely to be upper limits for such species.

There is evidence that many people do not use their bathroom fans when showering and certainly not to the extent that ventilation rates would be as high as 9 h^−1^.^41^ In order to test model sensitivity to this factor, the model runs were repeated using a ventilation rate of 4.5 h^−1^. Under these conditions, limonene mixing ratios peaked at around 495 ppb. Higher values were sustained for longer as would be expected with lower ventilation rates. Peak formaldehyde reached similar values under both ventilation rates, but remained elevated for ~1 h longer than shown in Figure 6 at the lower ventilation rate.

### 3.5 Comparisons against a proof of concept real‐life study

The activity assumptions used in Table 2 were assessed during a real‐life shower study. Product classes of facewash, followed by shower gel, shampoo, and conditioner were used by participants following the medium unit activity assumptions with measurements made in real time using on‐line mass spectrometry of ambient air within the room. After drying off for three minutes, moisturizer and an aerosol deodorant were applied (facewash and deodorant activity assumptions detailed in Table S4). To support the assumption that VOC emission will change linearly based on the amount of product used, a single participant showered three times, using each of the low, medium, and high PCP usage amounts (g), where period of use stayed consistent at the central value assumption. The air in the room was sampled while the participant showered using PTR‐MS, and the concentration of limonene released was determined after normalizing to a standard limonene calibrant.

**Figure 7:**
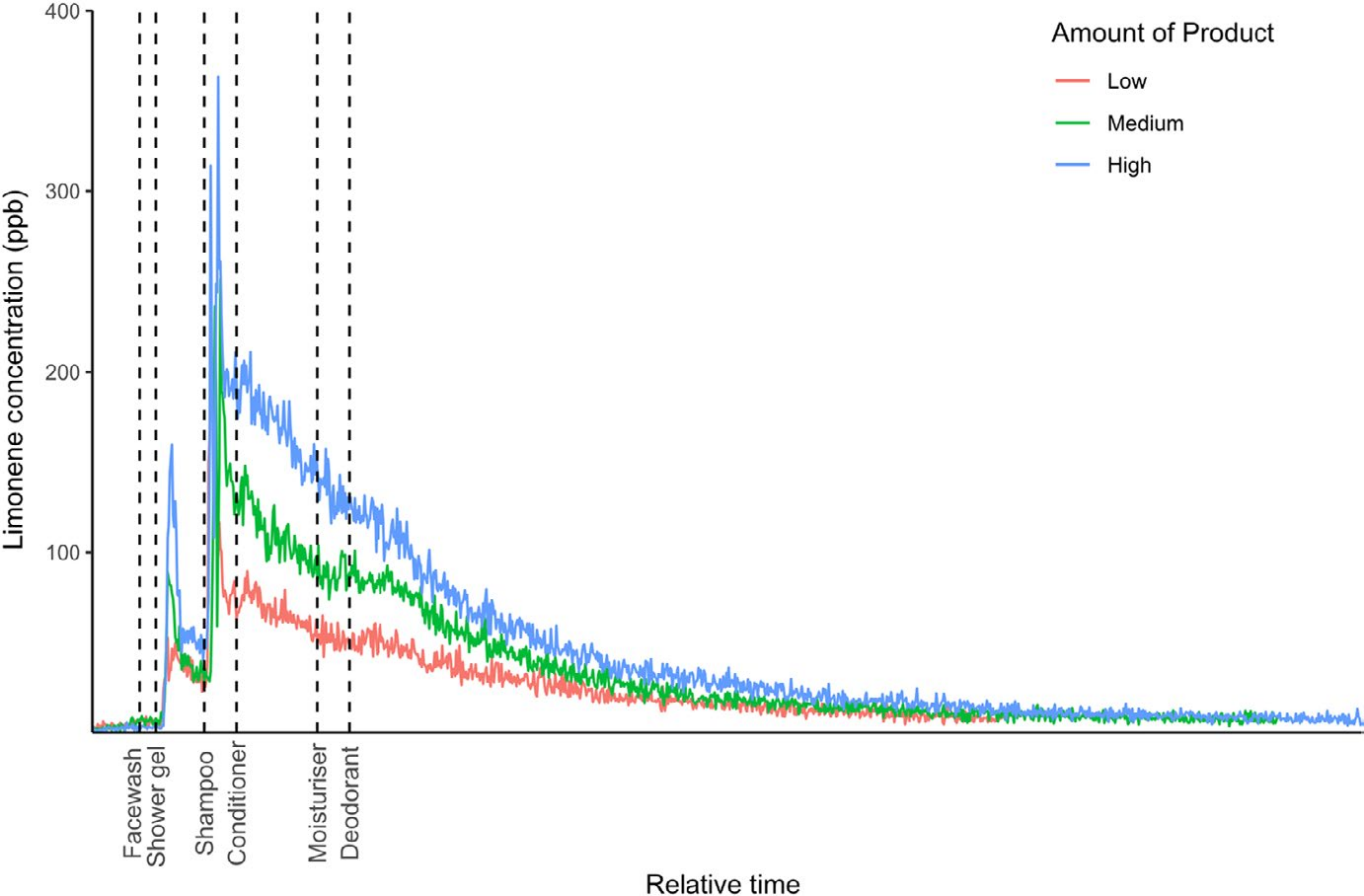
Mixing ratio of limonene measured during the real‐life shower study in low, medium, and high amount use scenarios

Figure 7 shows that scaling the amount of PCP used directly changed the limonene concentration in a linear fashion. Clearly, these are very limited experimental data. However, we include them to provide independent reassurance that the emission values calculated bottom‐up in this study and then included in the INDCM simulations, generate concentrations that are within an order of magnitude of those generated when the same quantities of PCP materials are used in the real‐world.

## 4 CONCLUSIONS

Online mass spectrometry methods have provided a straightforward method to screen for VOC composition and emission amount in a range of different VOC‐containing non‐aerosol personal care products. While every product has a unique composition, simplified profiles could be reported using seven common VOCs found in most of the samples screened (four alcohols and two siloxanes, and the lumped value for limonene to represent all monoterpenes). Overall, we find that amounts of individual VOCs released vary considerably between products, but are in the range of a few milligrams to a few grams of each VOC from each product per person per year. Shower gels and liquid foundation were found to have the highest rates of VOC emissions, dominated by limonene (representing all monoterpenes) for the former and D5 cVMS for the latter.

Few countries have a detailed and speciated emissions inventory for VOCs that is constructed at a sufficient level of granularity such that VOCs deriving from non‐aerosol PCPs can be uniquely identified. The UK National Atmospheric Emissions Inventory does report at this level of detail, and this is compared to national emission estimates made for each VOC based on the bottom‐up data collected here. Four of the seven VOCs in the simplified emissions profile do not have non‐aerosol PCP emissions associated with them in the NAEI, and in general, NAEI emissions are considerably lower than would be estimated using the bottom‐up figures. The most significant mass emissions per year are D5 cVMS (0.25 ktonne yr^−1^) and limonene (0.15 ktonne yr^−1^). Given annual VOC emissions for the UK are of the order ~800 ktonne yr^−1^, it is clear that the under‐representation of non‐aerosol PCPs in isolation in the NAEI is unlikely to introduce significant error into the estimates reported under the auspices of CLRTAP or NECD. However, PCPs are only one of many classes of domestic products that potentially release VOCs, most significantly aerosol‐based consumer products (eg, cosmetic, glues, car care) and household products (eg, fragrance, cleaning, pesticides), and some of these are also not currently reflected in inventory reporting.

As well as providing information for national emissions inventories, this work highlights the benefits of having product emissions rates in determining individual exposure to indoor air pollutants. On any one day, the exposure of an individual to air pollution is comprised of the sum of short‐lived, individual exposures to high concentrations of VOCs from activities such as showering, cooking, cleaning, and walking along a busy road, in addition to low levels of continuous exposure. While measurements both indoors and outdoors have provided us with a reasonable understanding of the latter process, we know very little about exposures from individual indoor activities. Personal exposure measurements are extremely time consuming to make and are typically only carried out on a few individuals at a time, posing issues for representativeness. PCP emission rates for VOCs therefore presents an opportunity to model, based on activity, personal exposure and to start to understand the relative importance of outdoor versus indoor exposure for different individuals.
