# Supplementary material for: Simplified speciation and atmospheric volatile organic compound emission rates from non‐aerosol personal care products
Source: Indoor Air. 2020 Feb 26;30(3):459–72. doi: 10.1111/ina.12652 (PMC7217173; doi:10.1111/ina.12652)
Supplement: Supplementary file 1 [file INA-30-459-s001.docx]

| **Monoterpene** | **Molar Mass** | **Removed Ions (m/z)** | | |
| --- | --- | --- | --- | --- |
|  |  | **H_3_O^+^** | **NO^+^** | **O_2_^+^** |
| 3-Carene | 136 | 81 | 92, 93, 135 | 80, 92, 94, 107, 121, 136 |
| Camphene | 136 | 81 | 92, 93, 94, 121, 166 | 80, 92, 107, 108, 121, 136 |
| α-Pinene | 136 | 81 | 92, 93 | 80, 92, 107, 121, 136 |
| β-Pinene | 136 | 81 | 92, 93 | 69, 80, 92, 107, 121, 136 |
| Limonene | 136 | 51, 69, 77 | 88 | 94, 107, 121, 136. 137 |
| Myrcene | 136 | 69, 81, 95 | 92, 93 | 69, 80, 92, 94, 121, 136 |
| α/β-Ocimene | 136 | 57, 69, 81, 95 | 92, 93 | 68, 80, 92, 94, 107, 121, 136 |
| Carvone | 150 |  | 150 | 81, 106, 108, 150 |
| Thymol | 150 |  | 150 | 135, 150 |
| Citral | 152 | 95 | 94, 151, 152, 182 |  |
| Verbenol | 152 | 81, 135, 139 | 93, 94, 134, 150, 152, 182 | 59, 94, 109, 152 |
| Camphor | 152 |  | 151, 152, 182 | 80, 81, 95, 108, 110, 152 |
| Eucalyptol | 154 | 155 | 184 | 108, 111, 154 |
| Borneol | 154 |  | 153 | 95, 110, 154 |
| Citronellal | 154 | 59, 81, 95, 155 | 111, 112, 125 | 43, 84, 110, 111, 112, 121, 136, 139, 154 |
| Geraniol | 154 | 155 | 137 | 69, 123, 136 |
| Rose Oxide | 154 | 99 | 153 | 139 |
| Linalool | 154 | 81, 95 | 92, 96, 111 | 80, 83, 96, 121, 137 |
| Linalyl Acetate | 196 | 59, 81 | 80, 88 | 43, 59, 80 |

**Supplementary Information**

**SI Table 1**: Monoterpene ions removed from Figure 2, SI Figure 1, and SI Figure 2


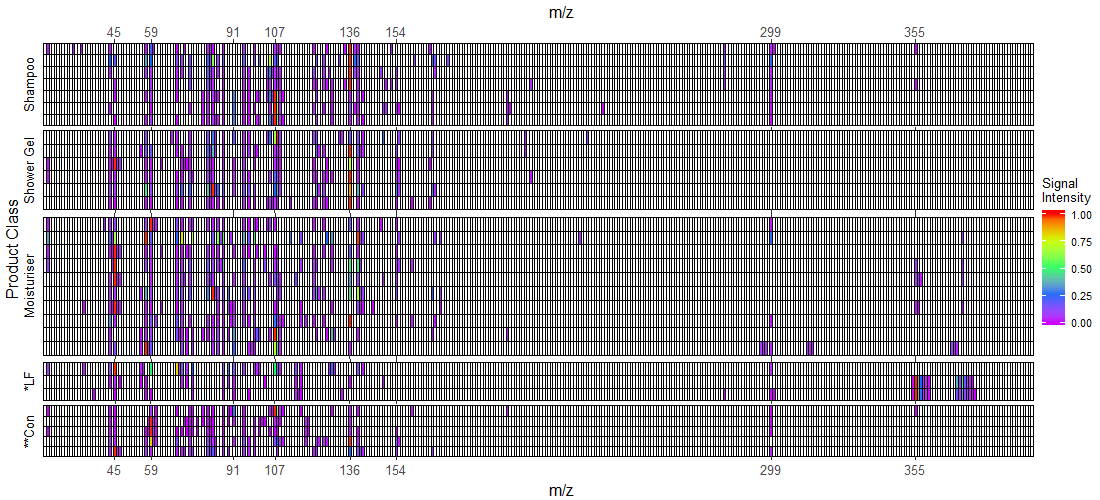


**SI Figure 1**: Visualisation of VOC emissions from PCPs based on NO^+^ ionisation. Data from each PCP sample is normalised to the maximum product ion intensity in that sample. Fragment ions are removed. *LF- Liquid Foundation, **Con-Conditioner


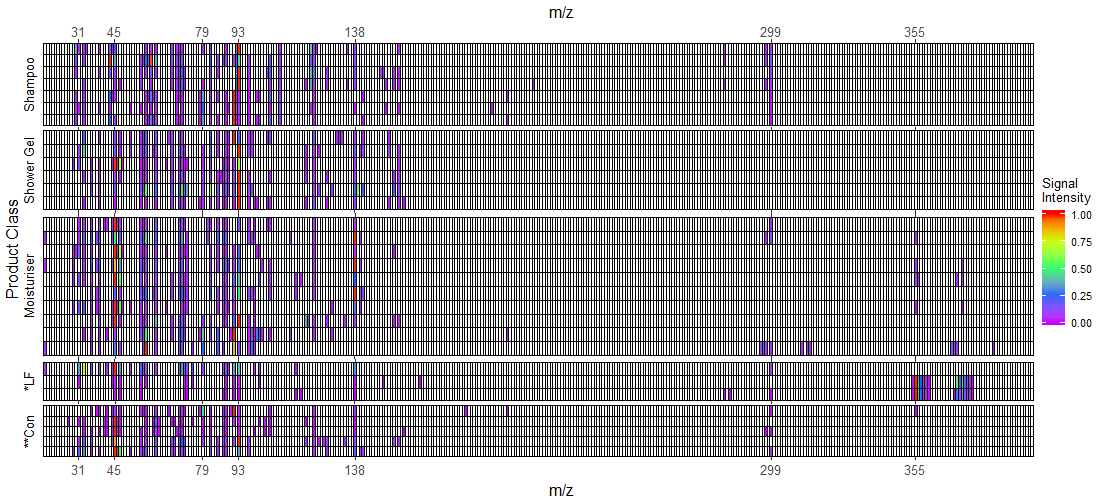


**SI Figure 2**: Visualisation of VOC emissions from PCPs based on O_2_^+^ ionisation. Data from each PCP sample is normalised to the maximum product ion intensity in that sample. Fragment ions are removed. *LF- Liquid Foundation, **Con-Conditioner

**SI Table 2**: Average concentrations of VOCs emitted for each non-aerosol PCP product during headspace analysis over a period of two hours (mg/L). These values were used to calculate the emission factors in Table 1

|  |  | |  |  |  |  |  |  |  |
| --- | --- | --- | --- | --- | --- | --- | --- | --- | --- |
|  | |  | 2-Propanol | Benzyl Alcohol | D4 | D5 | Ethanol | Limonene | Methanol |
| Shampoo | | 1 | 8.07 x10^-4^ | 1.55X10^-4^ | 1.96 x10^-3^ | 1.99 X10^-4^ | 5.74 x10^-5^ | 4.47 x10^-2^ | 7.22 x10^-5^ |
|  |  | 2 | 2.84 x10^-4^ | 9.48 x10^-4^ | 3.08 x10^-3^ | 3.20 x10^-4^ | 2.71 x10^-4^ | 6.88 x10^-3^ | 3.29 x10^-4^ |
|  |  | 3 | 1.02 x10^-4^ | 1.98 x10^-4^ | 1.38 x10^-3^ | 1.95 x10^-4^ | 9.13 x10^-5^ | 1.84 x10^-2^ | 1.56 x10^-4^ |
|  |  | 4 | 5.36 x10^-5^ | 6.38 x10^-5^ | 1.62 x10^-3^ | 2.07 x10^-4^ | 1.91 x10^-4^ | 1.81 x10^-2^ | 4.72 x10^-4^ |
|  |  | 5 | 8.53 x10^-4^ | 4.09 x10^-2^ | 1.50 x10^-3^ | 3.26 x10^-4^ | 1.28 x10^-4^ | 1.12 x10^-3^ | 8.99 x10^-4^ |
|  |  | 6 | 4.55 x10^-5^ | 3.12 x10^-2^ | 1.22 x10^-3^ | 2.69 x10^-4^ | 5.75 x10^-5^ | 5.42 x10^-3^ | 1.39 x10^-3^ |
|  |  | 7 | 1.04 x10^-4^ | 7.23 x10^-4^ | 1.53 x10^-3^ | 3.35 x10^-4^ | 3.57 x10^-4^ | 5.33 x10^-2^ | 1.38 x10^-4^ |
| Shower Gel | | 1 | 8.73 x10^-5^ | 1.37 x10^-2^ | - | - | 1.88 x10^-4^ | 3.32 x10^-3^ | 1.28 x10^-3^ |
|  |  | 2 | 2.45 x10^-3^ | 5.18 x10^-4^ | - | - | 4.53 x10^-4^ | 9.30 x10^-2^ | 7.73 x10^-3^ |
|  |  | 3 | 5.32 x10^-5^ | 5.12 x10^-5^ | - | - | 7.60 x10^-3^ | 1.10 x10^-2^ | 5.83 x10^-5^ |
|  |  | 4 | 3.70 x10^-5^ | 5.07 x10^-4^ | - | - | 1.22 x10^-4^ | 4.87 x10^-3^ | 2.06 x10^-4^ |
|  |  | 5 | 6.46 x10^-5^ | 1.99 x10^-4^ | - | - | 6.03 x10^-5^ | 1.10 x10^-3^ | 6.72 x10^-5^ |
|  |  | 6 | 4.37 x10^-5^ | 2.91 x10^-5^ | - | - | 6.97 x10^-5^ | 3.10 x10^-2^ | 1.41 x10^-4^ |
| Moisturiser | | 1 | 2.31 x10^-3^ | 3.72 x10^-5^ | - | 1.48 x10^-2^ | 4.92 x10^-4^ | 1.01 x10^-3^ | 3.05 x10^-4^ |
|  |  | 2 | 1.97 x10^-5^ | 3.19 x10^-6^ | - | 9.94 x10^-3^ | 8.02 x10^-5^ | 4.05 x10^-5^ | 6.46 x10^-5^ |
|  |  | 3 | 2.23 x10^-4^ | 1.94 x10^-5^ | - | 1.11 x10^-2^ | 6.47 x10^-3^ | 2.11 x10^-3^ | 1.89 x10^-4^ |
|  |  | 4 | 1.61 x10^-5^ | 2.06 x10^-5^ | - | 1.53 x10^-2^ | 2.30 x10^-4^ | 2.39 x10^-4^ | 3.67 x10^-5^ |
|  |  | 5 | 6.30 x10^-5^ | 7.64 x10^-4^ | - | 1.27 x10^-1^ | 2.34 x10^-3^ | 1.75 x10^-3^ | 1.60 x10^-4^ |
|  |  | 6 | 3.06 x10^-5^ | 8.78 x10^-5^ | - | 1.06 x10^-2^ | 4.56 x10^-5^ | 5.72 x10^-4^ | 6.61 x10^-5^ |
|  |  | 7 | 5.41 x10^-5^ | 7.75 x10^-5^ | - | 3.50 x10^-2^ | 2.39 x10^-3^ | 2.19 x10^-5^ | 8.73 x10^-4^ |
|  |  | 8 | 3.02 x10^-4^ | 9.07 x10^-4^ | - | 1.61 x10^-2^ | 7.52 x10^-5^ | 3.98 x10^-3^ | 6.06 x10^-5^ |
|  |  | 9 | 7.66 x10^-4^ | 1.44 x10^-2^ | - | 2.84 x10^-2^ | 8.49 x10^-5^ | 8.51 x10^-5^ | 1.71 x10^-4^ |
|  |  | 10 | 4.83 x10^-4^ | 4.93 x10^-3^ | - | 2.79 x10^-2^ | 2.59 x10^-4^ | 1.15 x10^-4^ | 1.31 x10^-4^ |
| Liquid Foundation | | 1 | - | - | - | 1.97 x10^-4^ | 4.65 x10^-4^ | 1.18 x10^-5^ | - |
|  |  | 2 | - | - | - | 4.69 x10^-1^ | 5.38 x10^-4^ | 2.08 x10^-4^ | - |
|  |  | 3 | - | - | - | 4.79 x10^-1^ | 2.93 x10^-5^ | 1.28 x10^-5^ | - |
| Conditioner | | 1 | 4.62 x10^-4^ | 1.03 x10^-2^ | 3.96 x10^-4^ | 1.27 x10^-2^ | 3.58 x10^-5^ | 3.60 x10^-4^ | 1.47 x10^-5^ |
|  |  | 2 | 5.78 x10^-2^ | 2.74 x10^-3^ | 1.09 x10^-3^ | 4.32 x10^-4^ | 4.00 x10^-5^ | 2.39 x10^-3^ | 1.56 x10^-3^ |
|  |  | 3 | 2.77 x10^-2^ | 7.48 x10^-5^ | 1.00 x10^-3^ | 4.29 x10^-4^ | 3.27 x10^-5^ | 1.15 x10^-3^ | 6.73 x10^-4^ |
|  |  | 4 | 2.05 x10^-3^ | 4.64 x10^-4^ | 5.50 x10^-4^ | 3.30 x10^-4^ | 5.30 x10^-5^ | 5.60 x10^-3^ | 4.68 x10^-4^ |
|  |  | 5 | 4.24 x10^-5^ | 7.90 x10^-5^ | 7.52 x10^-4^ | 2.57 x10^-4^ | 1.87 x10^-3^ | 1.11 x10^-3^ | 5.12 x10^-4^ |

**SI Table 3**: Annualised estimates of VOC emissions per person from selected non-aerosol personal care products based on the median emission rate product in each class.

|  | **Annualised Emissions (g person**^-1^ **year**^-1^**)** | | | | | | | |
| --- | --- | --- | --- | --- | --- | --- | --- | --- |
|  | 2-Propanol | Benzyl Alcohol | D4 | D5 | Ethanol | Limonene | Methanol |  |
| Shampoo | 4.5 x10^-4^ | 3.0 x10^-3^ | 6.7 x10^-3^ | 1.1 x10^-3^ | 5.7 x10^-4^ | 7.6 x10^-2^ | 1.3 x10^-3^ |  |
|  | 1.1 x10^-2^ | 7.1 x10^-2^ | 1.6 x10^-1^ | 2.6 x10^-2^ | 1.4 x10^-2^ | 1.8 | 3.2 x10^-2^ |  |
|  | 1.3 x10^-1^ | 8.1 x10^-1^ | 1.9 | 3.1 x10^-1^ | 1.6 x10^-1^ | 2.1 x10^1^ | 3.7 x10^-1^ |  |
| Shower Gel | 2.1 x10^-3^ | 1.3 x10^-2^ | - | - | 5.7 x10^-3^ | 3.0 x10^-1^ | 6.4 x10^-3^ |  |
|  | 2.0 x10^-2^ | 1.2 x10^-1^ | - | - | 5.3 x10^-2^ | 2.8 | 5.9 x10^-2^ |  |
|  | 1.3 x10^-1^ | 7.8 x10^-1^ | - | - | 3.6 x10^-1^ | 1.8 x10^1^ | 4.0 x10^-1^ |  |
| Moisturiser | 6.2 x10^-6^ | 3.6 x10^-6^ | - | 2.0 x10^-5^ | 1.1 x10^-5^ | 1.7 x10^-5^ | 6.5 x10^-6^ |  |
|  | 5.9 x10^-3^ | 3.4 x10^-3^ | - | 1.9 x10^-2^ | 1.0 x10^-2^ | 1.7 x20^-1^ | 6.2 x10^-3^ |  |
|  | 8.3 x10^-1^ | 4.8 x10^-1^ | - | 2.6 | 1.4 | 2.3 | 8.7 x10^-1^ |  |
| Liquid Foundation | - | - | - | 1.1 | 9.9 x10^-4^ | 2.9 x10^-5^ | - |  |
|  | - | - | - | 1.9 x10^1^ | 1.8 x10^-2^ | 5.3 x10^-4^ | - |  |
|  | - | - | - | 4.4 x10^2^ | 4.2 x10^-1^ | 1.2 x10^-2^ | - |  |
| Conditioner | 1.3 x10^-2^ | 2.9 x10^-3^ | 4.9 x10^-3^ | 2.7 x10^-3^ | 2.6 x10^-4^ | 7.3 x10^-3^ | 3.4 x10^-3^ |  |
|  | 3.1 x10^-1^ | 7.1 x10^-2^ | 1.2 x10^-1^ | 65 x10^-2^ | 6.1 x10^-3^ | 1.8 x10^-1^ | 8.0 x10^-2^ |  |
|  | 3.6 | 8.2 x10^-1^ | 1.4 | 7.6 x10^-1^ | 7.1 x10^-2^ | 2.1 | 9.4 x10^-1^ |  |

**SI Table 4**: PCP in-use consumption scenarios / activity levels for facewash and aerosol deodorant employed during shower study

| **Product Class** |  | **PCP Used in each Unit of Activity(g)** | **Period of Use (s)** | **Unit Activity**  **(s g)** |
| --- | --- | --- | --- | --- |
| Facewash | L | 0.5 | 30 | 15 |
|  | M | 2 | 60 | 120 |
|  | H | 4 | 120 | 480 |
| Deodorant | L | - | 2 | - |
|  | M | - | 4 | - |
|  | H | - | 8 | - |

For deodorant there is no Unity of Activity as an aerosol cannot be weighted out. Therefore product use was controlled by time only as the aerosol will release the same amount of product each time it is used.
